# Improving EEG-Based Motor Imagery Classification for Real-Time Applications Using the QSA Method

**DOI:** 10.1155/2017/9817305

**Published:** 2017-12-03

**Authors:** Patricia Batres-Mendoza, Mario A. Ibarra-Manzano, Erick I. Guerra-Hernandez, Dora L. Almanza-Ojeda, Carlos R. Montoro-Sanjose, Rene J. Romero-Troncoso, Horacio Rostro-Gonzalez

**Affiliations:** ^1^Laboratorio de Sistemas Bioinspirados, Departamento de Ingeniería Electrónica, DICIS, Universidad de Guanajuato, Carr. Salamanca-Valle de Santiago Km. 3.5 + 1.8 Km., 36885 Salamanca, GTO, Mexico; ^2^Laboratorio de Procesamiento Digital de Señales, Departamento de Ingeniería Electrónica, DICIS, Universidad de Guanajuato, Carr. Salamanca-Valle de Santiago Km. 3.5 + 1.8 Km., 36885 Salamanca, GTO, Mexico; ^3^Cuerpo Académico de Telemática, DICIS, Universidad de Guanajuato, Carr. Salamanca-Valle de Santiago Km. 3.5 + 1.8 Km., 36885 Salamanca, GTO, Mexico; ^4^Departamento de Arte y Empresa, DICIS, Universidad de Guanajuato, Carr. Salamanca-Valle de Santiago Km. 3.5 + 1.8 Km., 36885 Salamanca, GTO, Mexico; ^5^Departamento de Ingeniería Electrónica, DICIS, Universidad de Guanajuato, Carr. Salamanca-Valle de Santiago Km. 3.5 + 1.8 Km., 36885 Salamanca, GTO, Mexico; ^6^Neuroscientific System Theory, Department of Electrical and Computer Engineering, Technical University of Munich, Munich, Germany

## Abstract

We present an improvement to the quaternion-based signal analysis (QSA) technique to extract electroencephalography (EEG) signal features with a view to developing real-time applications, particularly in motor imagery (IM) cognitive processes. The proposed methodology (iQSA,* improved* QSA) extracts features such as the average, variance, homogeneity, and contrast of EEG signals related to motor imagery in a more efficient manner (i.e., by reducing the number of samples needed to classify the signal and improving the classification percentage) compared to the original QSA technique. Specifically, we can sample the signal in variable time periods (from 0.5 s to 3 s, in half-a-second intervals) to determine the relationship between the number of samples and their effectiveness in classifying signals. In addition, to strengthen the classification process a number of boosting-technique-based decision trees were implemented. The results show an 82.30% accuracy rate for 0.5 s samples and 73.16% for 3 s samples. This is a significant improvement compared to the original QSA technique that offered results from 33.31% to 40.82% without sampling window and from 33.44% to 41.07% with sampling window, respectively. We can thus conclude that iQSA is better suited to develop real-time applications.

## 1. Introduction

In the last few years, interest in inferring information from the human brain stemming from cognitive thoughts by means of electroencephalography (EEG) has expanded to various disciplines such as neuroscience, robotics, computational science, physics, and mathematics. Research in these areas tends to revolve around the development of new communication and control technologies based on brain-computer interface (BCI) devices to support people with severe neuromuscular conditions in ways that can enable them to express their wishes or use devices as neuroprosthetics [1], wheelchairs [2, 3], control a cursor on a computer screen [4], or even a robot [5, 6]. Wolpaw et al. [7] argue that BCIs “give their users communication and control channels that do not depend on the brain's normal output channels of peripheral nerves and muscles.” In other words, BCI devices establish a communication channel between the individual and a component (electromechanical devices, robots, software applications, etc.) to control it by means of brain activity generated by the user to carry out some intended action [8]. To that effect, the user must call upon those actions by means of a brain strategy known as motor imagery.

Motor imagery (MI) is a conscious process defined as a mental simulation of a particular movement [9]. The motor imagery is endowed with the same functional relationship to the imagined or represented movement and the same causal role in the generation of the movement in question [10]. In other words, MI is related to the intention and preparation of movements, whereby the subject imagines carrying out a particular action without making any real movements. This has led to studies using motor imagery to decipher processes that precede the execution of an action. For instance, Bai et al. [11] claim that mental practice using motor imagery of limb movement may facilitate motor recovery in persons who have experienced cerebrovascular injuries. In addition, McFarland et al. [12] conducted a comparative analysis of EEG topographies associated with actual hand movements and imagined hand movements, concluding that motor imagery plays an important role in EEG-based communication and suggesting that mu and beta rhythms might provide independent control signals.

Similarly, there have been studies focusing on MI support and BCI systems, proposing algorithms for feature extraction and classification. For instance, Pfurtscheller et al. [13] studied the reactivity of mu rhythms associated with the imagination of hand, foot, and tongue movements with 60 EEG electrodes in nine able-bodied subjects (with a 66.16% performance rate). In turn, Aghaei et al. [14] argue for the use of the separable common spatiospectral patterns (SCSSP) method to extract discriminant spatiospectral EEG features and a Laplacian filter of data set V of BCI competition III with the following mental imagery tasks: left-hand movement, right-hand movement, and generation of words beginning with a random letter involving 3 subjects. As for Gao et al. [15], they conducted EEG signal analysis during left-hand movements, right-hand movements, and resting with 10 subjects using the Kolmogorov complexity to extract the features and an AdaBoost multiclass classifier, achieving a 79.5% accuracy rate. In a separate study, Schlögl et al. [16] conducted a comparative study involving four classifiers to determine the global separability of data in relation to four different MI tasks with 5 subjects, modelling the EEG signal by means of an adaptive autoregressive (AAR) process whose parameters were extracted through Kalman filtering. Trad et al. [17] used empirical mode decomposition (EMD) and band power (BP) to extract EEG signals and classify MI in experiments involving 10 subjects aged 22–35 as they imagined left-hand and right-hand movements. Choi and Cichocki [18] implemented a MI-based algorithm to control a wheelchair using spatial filters to extract the features by means of a common spatial pattern (CSP) method and the linear SVMs to classify feature vectors. Three healthy men participated in the experiments where they had to imagine clenching the right hand, squeezing the left hand, and walking. In [19] results of tests conducted with a 74% accuracy rate to control a robot indoors on the basis of three mental states are presented. In particular, there are several studies focusing on the development of processing techniques, feature extraction, and classification to improve the BCI systems. In Tables 1 and 2, we present the most commonly used algorithms for these tasks from Lotte' et al. works [20]. However, we only extracted information related to our work, that is, motor imagery activities (imagined movement of the left hand, imagined movement of the right hand, imagined movement of the foot, imagined movement of the tongue, relaxation, and mental calculation). These works use different algorithms in the classification stages, such as SVM, KNN, LDA, MLP, HMM, Gaussian classifier, and Bayes quadratic, including combinations of these, which result in most studies in acceptable performance rates. However, most experiments are performed on a small number of subjects, which returns a low number of trials per session. In the feature extraction stage, most techniques used to analyze EEG signals extract information within the frequency or time-frequency domain, which may lead to information loss when information is transformed. In addition, they require noise-elimination filters and frequency band localization to identify the patterns of motor imagery.

On the other hand, in [21], the design of a motor imagery experiment was reported based on three mental processes: arrow moving to the left, arrow moving to the right, and waiting time, lasting 5 seconds per image. This study analyzed EEG signals with the implementation of the quaternion-based signal analysis (QSA). The quaternion-based signal analysis (QSA) method is a technique that uses EEG signals within the time domain because it is based on quaternion algebra. The use of quaternion algebra with the QSA technique makes it possible to describe signals within the time domain by means of rotations and orientations of 3D objects and represent multichannel EEG signals as a single entity, preventing data ambiguity and producing a more accurate representation doing fewer calculations than are needed with other techniques. The offline analysis was conducted in the feature extraction phase considering the total number of samples in each class, which produced an 84.92% accuracy rate. However, while the analysis of offline signals is convenient and efficient, offline analysis results may not generalize their performance to online applications. In the case of the QSA method, the online analysis obtained was just 33.31% considering window sizes of 0.5 seconds.

Thus, this paper presents an improvement to the QSA method that we shall call* improved quaternion-based signal analysis* (iQSA) for use in the feature extraction and classification phases, whose contribution consists in providing a technique for use in real-time applications, focusing on analyzing EEG signals online reducing the sample sizes needed to a tenth of the ones required by QSA, resulting in a faster response and fewer delays to improve execution times in real-time actions. The experiment involved using an Emotiv-Epoc device to acquire the brain signals from the motor and visual regions of the cerebral cortex. Similarly, during the training and validation phases, the EEG signal database was strengthened by adding a greater number of subjects and by combining decision trees in the classification phase based on use of the boosting technique.

## 2. Materials and Methods

### 2.1. Quaternions

Quaternions were proposed in 1843 by Hamilton [22], as a set of four constituents (a real and three imaginary components) as follows: *q* = *w* + *ix* + *jy* + *kz*, where *w*, *x*, *y*, *z* ∈ *ℝ* and *i*, *j*, *k* are symbols of three imaginary quantities known as imaginary units. These units follow these rules:

(1) i2=j2=k2=ijk=−1ij=k,jk=i,ki=jji=−k,kj=−i,ik=−j.

A quaternion can be described as

(2) $$\begin{array}{c} q=\left(\;s+a\;\right),\;a=\left(x,y,z\right), \end{array}$$

where *s* and** a** are known as the quaternion's scalar and vector, respectively. When *s* = 0, *q* is known as pure quaternion.

Based on the expanded Euler's formula, the rotation for quaternion around the axis *n* = [*nx*, *ny*, *nz*] by angle theta is defined as follows (see [21, 45] for further details).

A rotation of angle *θ* around a unit vector **a** = [*a*_*x*_, *a*_*y*_, *a*_*x*_] is defined as follows:

(3) $$\begin{array}{c} q=\cos\left(\;\frac{\theta }{2}\;\right)+\left(\;a_{x}\cdot i+a_{y}\cdot j+a_{z}\cdot k\;\right)\sin\left(\;\frac{\theta }{2}\;\right). \end{array}$$

Furthermore, the operation to be performed on a vector** r** to produce a rotated vector** r**′ is

(4) r′=qrq−1=cos⁡θ2+asin⁡θ2·rcos⁡θ2−asin⁡θ2.

Equation (4) is a useful representation that makes the rotation of a vector easier. We can see that** r** is the original vector, **r**′ is the rotated quaternion, and *q* is the quaternion that defines the rotation.

### 2.2. iQSA Algorithm

The iQSA is a method that improves the performance and precision of the QSA method, which is a technique to analyze EEG signals for extracting features based on rotations and orientations by means of quaternion algebra. With iQSA, we can conduct real-time signal analysis as the signals are being acquired, to difference of QSA method who performs an offline analysis.

The iQSA approach consists of three modules: quaternion, classification, and learning, which are described as follows.

(1) Quaternion module: in this module a features matrix (*M*) is defined from the description of the quaternion *q* and a vector *R*, where each of them corresponds to an array of 4 and 3 EEG channels, respectively. This module is divided into three steps, which are described as follows:
  (a) Sampling window: here, we define the sample size (*ns*) to be analyzed and a displacement of the window in the signal (*t*_disp). That is, the iQSA method performs the sampling by means of superposing, producing a greater number of samples to reinforce the learning stage of the algorithm.
  (b) Calculate rotation and module: then, a rotated vector *q*_rot_ is calculated using the quaternion *q* and vector *R*, where *q* is a *m*-by-4 matrix containing *m* quaternions and *R* is an *m*-by-3 matrix containing *m* quaternions displaced on the basis of a *dt* value. Later, the modulus is applied to the quaternion *q*_rot_ resulting in the vector *q*_mod_.
  (c) Building an array of features: finally, the array *q*_mod_ is used to form a matrix with *M*_*i*,*j*_ features, where *i* corresponds to the analyzed segment and *j* is one of the 4 features to be analyzed using the equations included in Table 3, that is,* mean *(*μ*)*, variance *(*σ*^2^)*, contrast *(con), and* homogeneity *(*H*).
Equation (5) shows matrix *M* with its features vector. In this matrix rows correspond to samples and columns to features.
  (5) $$\begin{array}{c} M=\left[\begin{array}{cccc} \mu_{1} & \sigma_{1}^{2} & {con}_{1} & H_{1}\\ \mu_{2} & \sigma_{2}^{2} & {con}_{2} & H_{2}\\ \vdots & \vdots & \vdots & \vdots \\ \mu_{i} & \sigma_{i}^{2} & {con}_{i} & H_{i} \end{array}\right]. \end{array}$$
(2) Classification module: the aim of this module is to create a combination of models to predict the value of a class according to its characteristics; to do this we use the boosting method adapted to the QSA model. To be more specific, we combine ten decision trees and a weight is obtained for each of them, which will be used during the learning to obtain the prediction by a majority rule. Here, we take 70% of samples from *M*, of which the 80% are used for training and the rest for validation as follows:
  (a) Training: the subset of training data (80% samples) is assessed, using the models of decision trees to obtain a matrix with accurately classified samples (*G*) and a matrix with inaccurately classified samples (*B*). The learning of each tree is done by manipulating the training data set and partitioning the initial set in several subsets according to the classification results obtained; that is, a new subset is formed, generated, and created with the accurately classified samples, twice that number of inaccurately classified samples and the worst-classified samples from the original training data set. Later, when the trees of classification are created, these are used with the test data set to determine a weight in function of the accuracy of their predictions.
  (b) Validation: in this block we obtain a matrix with the reliability percentages given by the decision trees. The subset of validation data (20% samples) is assessed, using the decision trees to obtain an array with reliability values given by the processing of decision trees and the classes identified in the training process.
(3) Learning module: in this module, the subset of test data of *M* (30% samples) is assessed to obtain reliability values matrix (*R*) and the prediction by majority rule $\left(\hat{C}\right)$. That is, the learning process will be conducted assessing features matrix of test using the trees that have been generated in classification process. The final predictor comes from a weighted majority rule of the predictors from various decision trees. Equation (6) shows the recognition and error rates obtained in each of the prediction models. In this matrix RT corresponds to accuracy rate, ET corresponds to error rate, and *α* corresponds to reliability value of each decision tree.
  (6) $$\begin{array}{c} R=\left[\begin{array}{ccc} {RT}_{1} & {ET}_{1} & \alpha_{1}\\ {RT}_{2} & {ET}_{2} & \alpha_{2}\\ \vdots & \vdots & \vdots \\ {RT}_{i} & {ET}_{i} & \alpha_{i} \end{array}\right]. \end{array}$$

In Algorithm 1, we present the pseudocode for the main elements of the iQSA algorithm towards real-time applications.

#### 2.2.1. iQSA versus QSA Methods

**Figure 1:**
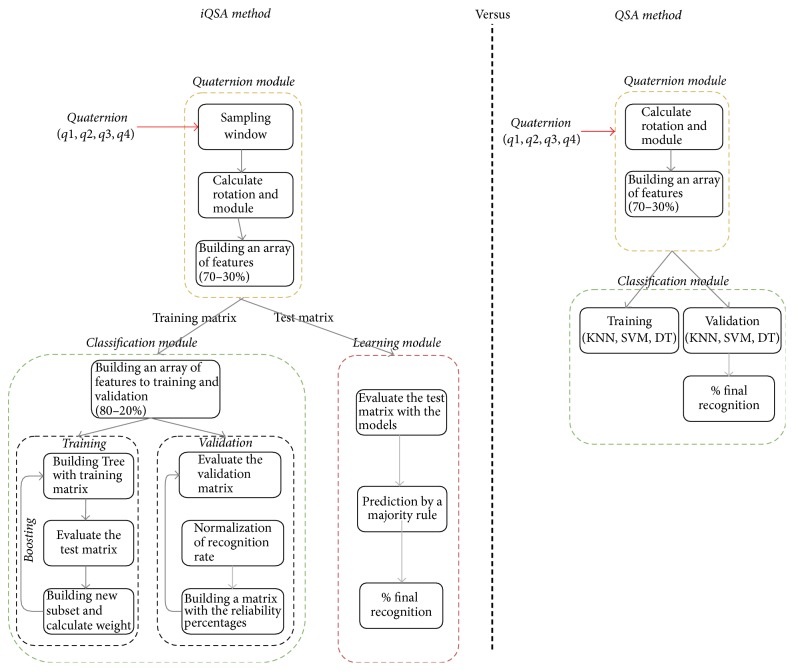
iQSA and QSA comparison.

In Figure 1, we show through block diagrams the main differences between the iQSA and QSA methods. It can be observed that QSA method considers quaternion and classification modules. On the other hand, iQSA method considers an improved classification module and one more for learning.

In Table 4, we present some of the main improvements made in the iQSA algorithm.

The QSA method, by its characteristics, is an excellent technique for the offline processing of EEG signals considering large samples of data and being inefficient when such data is small. This last becomes essential for online processing because the operations depend on the interaction in real time between the signals produced by the subject and the EEG signals translated with the aid of the algorithm. In this regard, the iQSA method considers small samples of data and creates a window with the superposition technique for a better data classification during feature extraction, strengthening the classification phase by means of the boosting technique.

**Figure 2:**
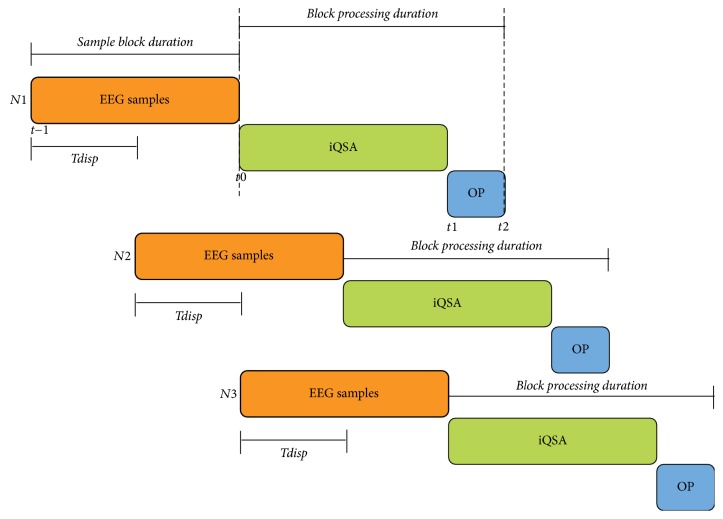
Timeline of events in the iQSA algorithm. Here, the iQSA performs the three aforementioned modules and OP represents the class obtained.

In this way, the signals produced by the iQSA algorithm affect the posterior brain signals, which in turn will affect the subsequent outputs of the BCI.  Figure 2 presents the time system diagram of iQSA, which indicates the timeline of events in the algorithm. It follows the process of three EEG data blocks (*N*_1_, *N*_2_, *N*_3_) obtained from the Emotiv-Epoc device. The processing block covers the analysis and processing of the data with the iQSA method until obtaining an output (OP), in this case, the class to which each *N*_*i*_ block belongs.

It is also possible to observe the start and duration of the next two sets of data, where the start of block *N*_2_ is displaced from the progress of block *N*_1_ by a time represented as *T*_disp_ between *t*_−1_ and *t*_0_; therefore, while the block *N*_1_ reaches the output at *t*_2_, the process of *N*_2_ continues executing until reaching its respective output.

### 2.3. BCI System

**Figure 3:**
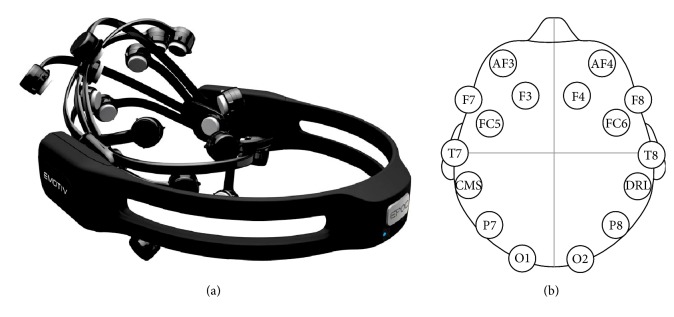
BCI system: (a) Emotiv-Epoc headset and (b) Emotiv-Epoc electrode arrangement.

A brain-computer interface is a system of communication based on neural activity generated by the brain. A BCI measures the activity of an EEG signal by processing it and extracting the relevant features to interact in the environment as required by the user. An example of this device is the Emotiv-Epoc headset (Figure 3(a)), a noninvasive mobile BCI device with a gyroscopic sensor, and 14 EEG channels (electrodes) and two reference channels (CMS/DRL) with a 128 Hz sample frequency. The distribution of sensors in the headset is based on the international 10–20-electrode placement system with two sensors as reference for proper placement on the head with channels labeled as AF3, F7, F3, FC5, T7, P7, O1, O2, P8, T8, FC6, F4, F8, and AF4 (Figure 3(b)).

An advantage of the Emotiv-Epoc device is its ability to handle missing values, very common and problematic when dealing with biomedical data.

### 2.4. Decision Trees and Boosting

Decision trees (DT) [46–48] are a widely used and easy-to-implement technique that offers high speed and accuracy rates. DT are used to analyze data for prediction purposes. In short, they work by setting conditions or rules organized in a hierarchical structure where the final decision can be determined following conditions established from the root to its leaves.

Recently, several alternative techniques have been presented to construct sets of classifiers whose decisions are combined in order to solve a task and to improve the results obtained by the base classifier. There exist two popular techniques to build sets: bagging [49] and boosting [50]. Both methods operate under a base-learning algorithm that is invoked several times using various training sets. In our case, we implemented a new boosting method adapted to QSA method, whereby we trained a number of weak classifiers iteratively so that each new classifier* (weak learner)* focuses on finding weak hypothesis (inaccurately classified). In other words, boosting calls the weak learner *w* times thus determining, at each iteration, a random subset of training samples by adding the accurately classified samples, twice that number of inaccurately classified samples and the worst-classified samples from the original training data set to form a new weak learner *w*.

As a result, inaccurately classified samples of the previous iteration are given an (*α*) weight in the next iteration, forcing the classifying algorithm to focus on data that are harder to classify in order to correct classification errors of the previous iteration. Finally, the reliability percentage of all the classifiers is added and a hypothesis is obtained by a majority vote, whose prediction tends to be the most accurate.

### 2.5. Channel Selection for iQSA

From the 14 electrodes that the Emotiv-Epoc device provides, we decided to perform an analysis on the electrodes located in three different regions of the cerebral cortex looking for those with better performance to form the quaternion: (1) electrodes located on the motor cortex, which generates neural impulses that control movements, (2) those on the posterior parietal cortex, where visual information is transformed into motor instructions [51], and (3) the ones on the prefrontal cortex, which appear as a marker of the anticipations that the body must make to adapt to what is going to happen immediately after [52].

In this regard, in Table 5 we present the performance for each of the sets of channels that were selected and further analyzed by the iQSA algorithm.

**Figure 4:**
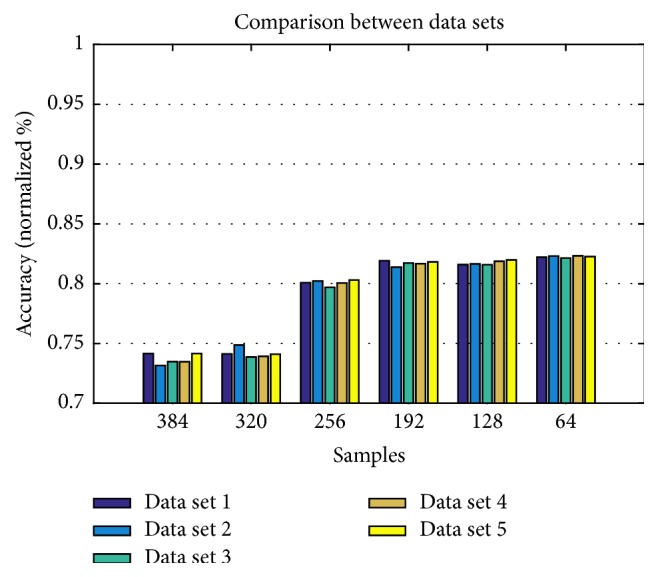
Comparison of data sets with different sample sizes. Data set 1: FC5, FC6, P7, P8; data set 2: F3, F4, FC5, FC6: data set 3, F3, F4, F7, F8; data set 4: F3, F4, P7, P8; data set 5: AF3, AF4, FC5, FC6.

Comparing the data sets shown in Figure 4, it is observed that all have a similar behavior in each sample size. For a 64-sample analysis, data sets 2 and 4 obtained 82.30% and 82.33%, respectively. The channels for data set 2 are related to the frontal and motor areas of the brain and the channels for data set 4 are related to the parietal and motor areas. From these results and due to the fact that we mainly focus on motor control tasks, set 2 (F3, F4, FC5, and FC6) has been chosen.

## 3. Experiment

As seen in earlier sections, one of the objectives of this paper is to analyze EEG signals towards real-time applications. Therefore, it is necessary to reduce the number of samples of EEG signals that have been acquired by subjecting them to a process based on the iQSA method to decode motor imagery activities, while keeping or improving accuracy results achieved with this technique. In this way, the experiment was designed to register EEG signals from several individuals and to identify three motor-imagery-related mental states (think left motion, think right motion, and waiting time).

### 3.1. Description of the Experiment

The experiment was conducted in three sessions: Session 1 involved motor imagery with tagged visual support (*V* + *L*), that is to say, an arrow with the word LEFT or RIGHT written on it. Session 2 involved motor imagery with visual (*V*) support only. In Session 3 the subject only received a tactile stimulus (*T*) to evoke motor imagery while keeping his or her eyes closed. In each session, the aim was to identify three mental states (think left motion, think right motion, and waiting time). The visual *V* and *VL* stimuli were provided using a GUI interface developed in Python 2.7 to show the movement of a red arrow for 5 seconds for each of the motor brain actions and a fixed cross at the center of the GUI to indicate a rest period for 3 seconds (the third brain action). The tactile stimulus was provided by touching the left/right shoulder of the individual to indicate the brain action to be performed. Each session lasted for 5 minutes and was done on separate days.

**Figure 5:**
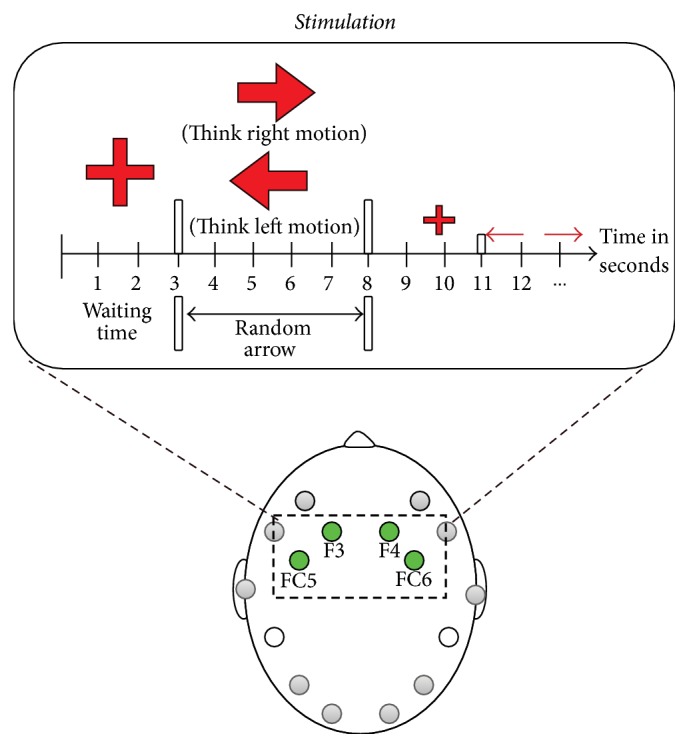
Methodology for activities in motor-cognitive experiments.

To start with, the participant was asked to sit on a comfortable chair in front of a computer screen. As the participant was given instructions regarding the test, an Emotiv-Epoc headset was placed on his or her head, making sure that each of the Emotiv device electrodes was making proper contact with the scalp (Figure 5). Once the participant was ready, he or she was asked not to make sudden body movements that could interfere with the signal acquisition results during the experiment.

The training paradigm consisted of a sequential repetition of cue-based trials (Figure 5). Each trial started with an empty blank screen; during the time *t* = 0 to *t* = 3 s a cross was displayed to indicate to the user that the experiment had started and that it was time to relax. Then at second 3 (*t* = 3 s) an arrow appearing for 5 s pointed either to the left or to the right. Each position indicated by the arrow instructed the subject to imagine left or right movement, respectively. The next trial started at *t* = 8 s with a cross. This process was repeated for 5 minutes, displaying the arrows 32 times and the cross 33 times within each run. Thus, the data set recorded for the three runs consisted of 96 trials.

### 3.2. iQSA Method Implementation

After data acquisition, the next stage consists in extracting EEG signal features in order to find the required classes, that is, think left motion, think right motion, and waiting time. To start with, the iQSA method was implemented to represent the four EEG signals within a quaternion and to carry out the feature extraction related to the stimuli presented. To that effect, data set 2 was prepared to assess their performance when time (and the number of samples) was reduced. Under these conditions, 3 seconds (384 samples), 2.5 seconds (320 samples), 2 seconds (256 samples), 1.5 seconds (192 samples), 1 second (128 samples), and 0.5 seconds (64 samples) were considered. Values 0, 1, and 2 were used to refer to the three mental activities: waiting time (0), think left motion (1), and think right motion (2).

**Figure 6:**
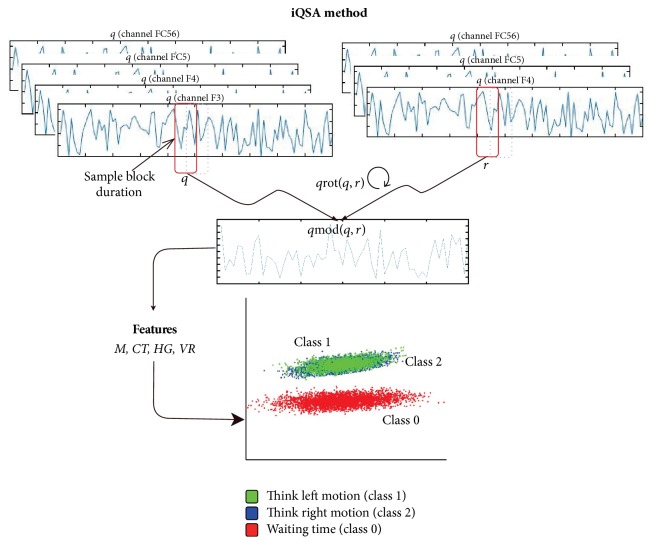
Graphic description of the iQSA method. *q*_rot_ represent the rotation of *q* with respect to *r*; *q*_mod_ is the module of *q*_rot_; class 1, class 2, and class 0 represent the motor activities evaluated, in this case “think left motion,” “think right motion,” and “waiting time,” respectively.

So, a segments matrix was obtained to detect sudden changes between classes for each data set. The segments matrix consisted of samples from 32 trials from left and right classes and samples from 33 trials for the waiting time class. In addition, the quaternion was created with the block signals proposed (F3, F4, FC5, and FC6), considering F3 as the scalar component and F4, FC5, and FC6 as the imaginary components (Figure 6). From the samples to be analyzed, several segments were created to generate the quaternion *q* and vector *R* with a displacement *dt* = 4 and thus obtain the rotation (*q*_rot_) and modulus (*q*_mod_).

Once the module was obtained, the* mean *(*μ*),* contrast *(con),* homogeneity *(*H*), and* variance *(*σ*^2^) features were calculated to generate the matrix *M* and the vector *c* with the required classes. Later, we returned to the current segment, and a displacement of 64 samples for the signal was effected to obtain the next segment.

Later,* M* was used in the processing stage using 70% of data from training and 30% from test; here each class has the same sample size and was randomly chosen. In the classification module, we generate the matrix *M*_1_ with the 80% from training data of *M* and *M*_2_ with the remaining 20% of the training data, which will be used for training and validation, where 10 training trees were created to force the algorithm to focus on the inaccurately classified data. Here, for each iteration we generate new subsets of samples with the double of inaccurately classified samples and fewer accurately classified samples, along with a reliability rate for each tree. Later, with matrix *M*_2_ the data were validated to obtain the percentage of reliability of each tree. Finally, with the remaining data of *M*, a prediction by majority rule is voted and a decision is reached based on the reliability rate of each classification tree.

## 4. Results

As said earlier, one of the aims of this study was to reduce the assessment time (sample size) without loss of the accuracy rate. Therefore, a comparison of the iQSA and QSA techniques was performed to show its behavior when the number of samples decreases.

### 4.1. Comparison between iQSA and QSA Methods

To evaluate and compare the performance of the iQSA online versus QSA offline algorithms, the same sets of data from the 39 participants were used, considering the different sample sizes (384, 320, 256, 192, 128, and 64 samples) as input for the algorithm.

**Figure 7:**
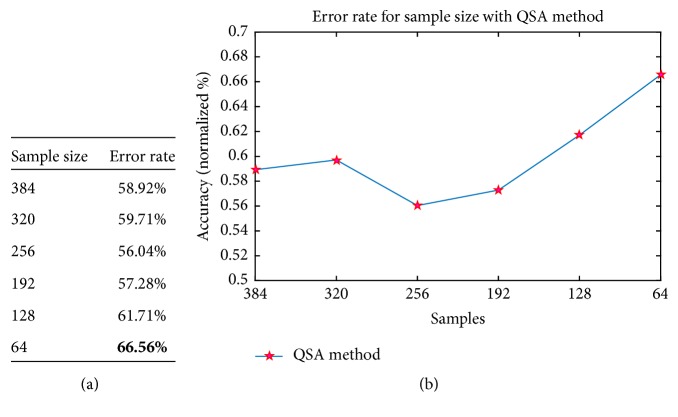
QSA method behavior. (a) Table of behavior of QSA method; (b) graphic of error rate for sample size.

In spite of the good performance rates reported with the QSA algorithm for offline data analysis, the classification results were not as good when the number of samples was reduced up to 64 samples as shown in Figure 7(a). Graphically, as can be seen, the error rate increases gradually when the sample size is reduced for analysis, reaching up to 66.56% error for a reading of 64 samples. In this way, it was necessary to make several adjustments to the algorithm, in such a way as to support an online analysis with small samples sizes, without losing information and sacrificing the good performance rates provided by the offline QSA algorithm.

Thus, the data of the 39 subjects were evaluated, considering three cases: (1) QSA method without window samples, (2) QSA method with window samples, and (3) iQSA proposed method. Comparing the results of Table 6, the precision percentages for the iQSA method range from 73.16% for 384 samples to 82.30%% for 64 samples, unlike the original QSA method whose percentage decreases to 33.31% for 64 samples in case 1 and 33.44% in case 2 although the sampling window has been implemented.

**Figure 8:**
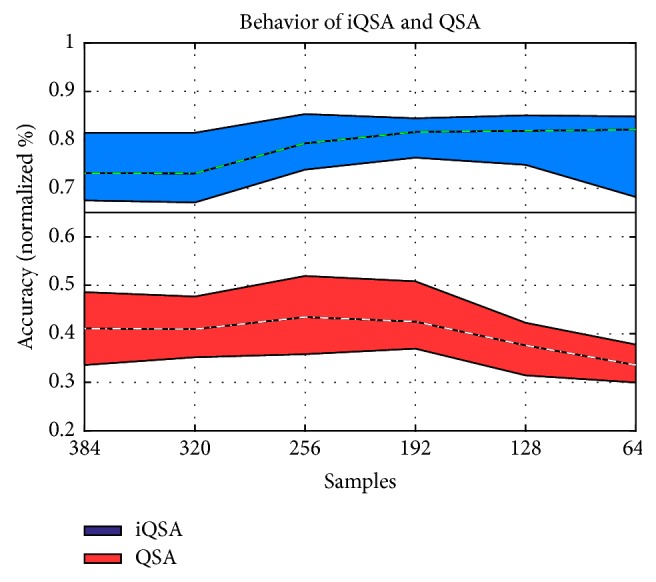
Behavior of the iQSA and QSA methods with different sample sizes.

Figure 8 shows the behavior of the iQSA technique in blue and QSA technique in red, where the mean, maximum, and minimum of each technique are shown. Comparing both techniques, it is observed that the data analyzed with the original QSA method drastically loses precision as the number of samples selected decreases. This was not the case for data sets using the iQSA technique whose precision did not vary too much when the number of samples for classification was reduced, improving even its performance.

### 4.2. iQSA Results

**Figure 9:**
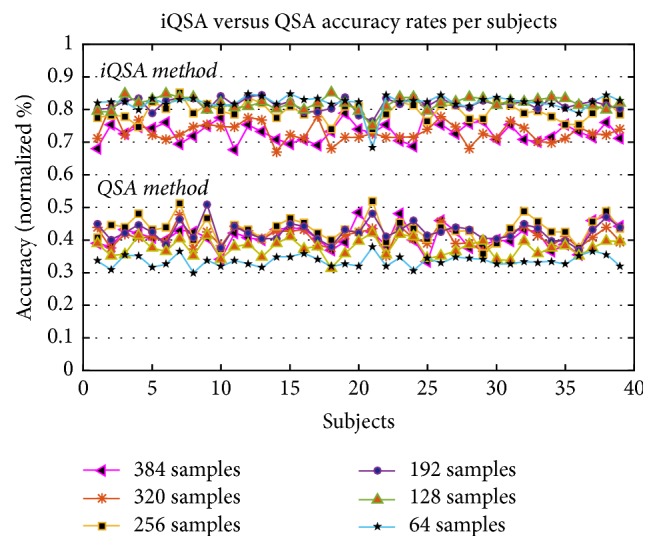
iQSA and QSA method performances per sample size and subjects.

Figure 9 shows the behavior of 39 participants under both techniques (iQSA and QSA), considering all the sample sizes. The values obtained using the iQSA method are better than values obtained with QSA method whose values are below 50%. Numerical results show that the iQSA method provides a higher accuracy over the original QSA method for tests with a 64-sample size.

Given the above results, a data analysis was conducted using 64 samples to identify the motor imagery actions. The performances obtained with the set of classifiers were compared using various assessment metrics, such as recognition rate (RT) and error rate (ET), and sensitivity (*S*) and specificity (Sp). The sensitivity metric shows that the classifier can recognize samples from the relevant class, and the specificity is also known as the real negative rate because it measures whether the classifier can recognize samples that do not belong to the relevant class.

(7) RT=#c ∣ c=c^#c

(8) ET=#c ∣ c≠c^#c

(9) Sd=#c ∣ c=d,c=c^#c ∣ c=d

(10) Spd=#c ∣ c≠d,c=c^#c ∣ c≠d.

As Table 7 shows, the highest accuracy percentage was 84.50% and the lowest 68.75%. In addition, the sensitivity average for class 0 (*S*_0_) associated with the waiting time mental state was 82.53%, and the sensitivity average for class 1 (*S*_1_) and class 2 (*S*_2_) associated with think left motion and think right motion was 81.07% and 81.65%, respectively, which indicates that the classifier had no problem classifying classes. In turn, the specificity rate for class 0 (Sp_0_) shows that 81.36% of the samples classified as negative were actually negative, while class 1 (Sp_1_) performed at 82.09% and class 2 (Sp_2_) at 81.80%.

To evaluate the performance of our approach, we have carried out a comparison with other methods such as FDCSP [53], MEMD-SI-BCI [54], and SR-FBCSP [55] using data set 2a from BCI IV [56] and the results are shown in Table 8. Our method shows a slight improvement (1.09%) compared to the SR-FBCSP method, which presents the best results of the three.

To analyze our results, we performed a significant statistical test making use of the STAC (Statistical Tests for Algorithms Comparison) web platform [57]. Here, we chose the Friedman test with a significance level of 0.10 to get a ranking of the algorithms and check if the differences between them are statistically significant.

Table 9 shows the Friedman test ranking results obtained with the *p* value approach. From such table, we can observe that our proposal gets the lowest ranking; that is, iQSA has the best results in accuracy among all the algorithms.

In order to compare whether the difference between iQSA and the other methods is significant, a Li post hoc procedure was performed (Table 10). The differences are statistically significant because the *p* values are below 0.10.

In addition, the classification shown in Table 8 is achieved by the iQSA in real time and compared to the other methods a prefiltering process is not required.

In Table 11, we show the time required for each of the tasks performed by the iQSA algorithm: (1) features extraction, (2) classification, and (3) learning. The EEG signal analysis in processes 1 and 2 was responsible for obtaining the quaternion from the EEG signals, learning trees, and training. Process 3 evaluates and classifies the signal based on the generated decision trees, as should be done in real-time analysis.

According to the experiment, for offline classification (processes 1 and 2), the processing time required was 0.3089 seconds. However, the time required to recognize the motor imagery activity generated by the subject was of 0.0095 seconds, considering that the learning trees were already generated, and even when the processing phase was done in real time, it would take 0.3184 seconds to recognize a pattern after the first reading. With this, our proposed method can be potentially used in several applications such as controlling a robot, manipulating a wheelchair, or controlling home appliances, to name a few.

**Figure 10:**
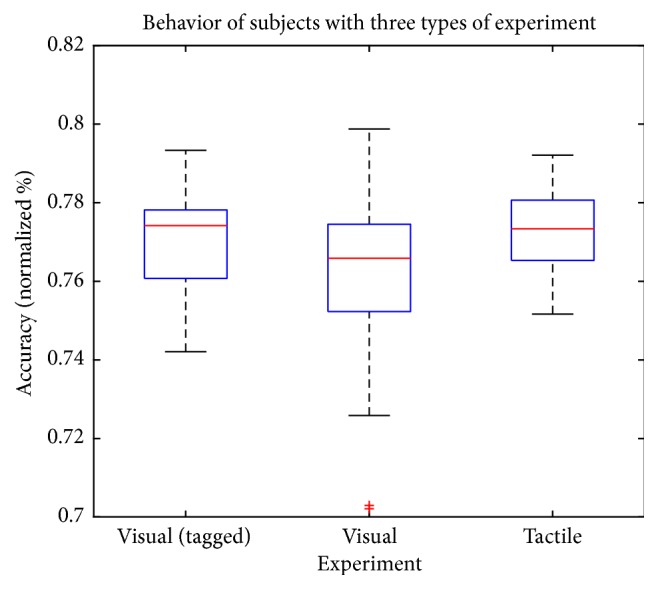
Graph of behavior of subjects with three types of experiments: tagged visual, visual, and tactile stimulation.

As said earlier, the experiment consisted of 3 sessions with each participant (visual tagged, visual, and tactile). The average accuracy obtained from the 3 experiments is between 76% and 78%.  Figure 10 shows results from the visual stimulation session, which produced the lowest rate at 76.63%, followed by tagged visual session which reached 76.98% and the tactile session 77.28%.

In Figure 10, it is shown that the mental activity generated with the aid of a tactile stimulus is slightly more accurate than visual and visual tagged stimuli where recognition is more imprecise and slow with visual stimulus.

To summarize, the results of tests conducted with 39 participants using this new method to classify motor imagery brain signals, 20 times with each participant, considering 70%–30% of the data have been presented and creating several subsets of 80–20% for the classification process. The average performance accuracy rate was 81.75% when using 10 decision trees in combination with the boosting technique at 0.5-second sampling rates. The results show that this methodology for monitoring, representing, and classifying EEG signals can be used for the purposes of having individuals control external devices in real time.

## 5. Conclusions

Feature extraction is one of the most important phases in systems involving BCI devices. In particular, feature extraction applied to EEG signals for motor imagery activity discrimination has been the focus of several studies in recent times. This paper presents an improvement to the QSA method known as iQSA, for EEG signal feature extraction with a view to using it in real time with mental tasks involving motor imagery. With our new iQSA method, the raw signal is subsampled and analyzed on the basis of a QSA algorithm to extract features of brain activity within the time domain by means of quaternion algebra. The feature vector made up of mean, variance, homogeneity, and contrast is used in the classification phase to implement a set of decision-tree classifiers using the boosting technique. The performance achieved using the iQSA technique ranged from 73.16% to 82.30% accuracy rates with readings taken between 3 seconds and half a second. This new method was compared to the original QSA technique, whose accuracy rates ranged from 40.82% to 33.31% without sampling window and from 41.07% to 33.34% with sampling window. We can thus conclude that iQSA is a promising technique with potential to be used in motor imagery recognition tasks in real-time applications.

## Figures and Tables

**Algorithm 1:**
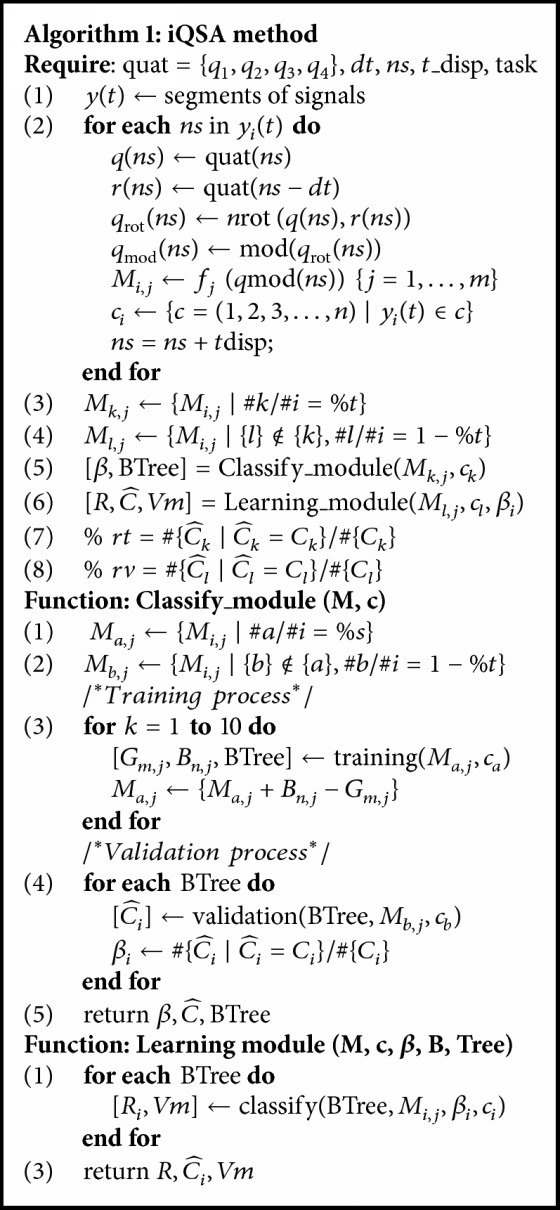
iQSA algorithm.

**Table 1:** Accuracy of classifier in motor imagery based BCI: multiclass. The classes are (T1) left imagined hand movements, (T2) right imagined hand movements, (T3) imagined foot movements, (T4) imagined tongue movements, (T5) relax (baseline), (T6) word generation starting with a letter specific, and (T7) mental calculation.

Dataset	Activity	Subjects	Trials	Filter	Feature	Classifier	Accuracy	References
BCI competition III	T1, T2, T3, T4	5	240 to 360	Yes	AAR	Linear SVM	63%	[16]
						KNN	41.74%	
						LDA	54.46%	
						Mahalanobis distance	53.50%	
	T1, T2, T6	3		Yes		Neuro-fuzzy algorithm S-dFasArt	89.04%	[23]
	T1, T2, T3, T4	3	240	Yes	CSP	CSP + SVM	79%	[24]
						CSP + SVM + KNN + LDA	69%	
						PCA + ICA + SVM	63%	
						CBN + SVM	91%	
	T1, T2, T6	3	12	Yes	SCSSP/LDA	FBCSP-NBPW	52.42 ± 6.94	[14]
						FBCSP-Lin	59.77 ± 9.97	
						SCSSP-NBPW	53.21 ± 6.94	
						SCSSP-Lin	59.33 ± 8.71	
	T1, T2, T3	5	280	Yes	CSP/ERD-ERS	BSSFO, SVM	75.46%	[25]

BCI competition IV	T1, T2, T3, T4	9	288	Yes	CSP	CSP + SVM	31%	[24]
						LDA + SVM	30%	
						CSP + SVM	29%	
						CBN + SVM	66%	
	T1, T2, T3, T4	9	288	Yes	SCSSP/LDA	LDA	85%	[14]
	T1, T2, T3, T4	9	288	Yes	CSP/ERD-ERS	BSSFO, SVM	80.26%	[25]

Other data sets	T1, T2, T3, T4	9	240	Yes	AAR, Kalman Filter	MDA	—	[13]
	T1, T2, T5	9	120	Yes		SMR, two-dimensional linear classifier	85%	[26]
	T1, T2, T3, T4	8	288	Yes	ICA, CSP	FDA	33%–84%	[27]
	T1, T2, T3	3	480	Yes	(SampEn)	SVM (RBF Kernel)	69.93%	[28]
	T1, T2, T5	33		Yes	AAR	PCA		[12]

Epoc	T1, T2, T3	3	100	Yes		SVM	66.16%	[29]
	T1, T2	5		Yes	ICA, CWT	Simple logistic, Meta, MLP	80.40%	[30]
	T1, T2, T4	1	75	Yes		Neural networks (PSO)	91%	[31]
	T1, T2, T3, T4, T7	3	40	Yes	BP	HMM	77.50%	[32]
	T1, T2, T3	3	30	Yes	BP	LDA	95%	[33]

**Table 2:** Accuracy of classifier in motor imagery based BCI: two classes. The classes are (T1) left imagined hand movements and (T2) right imagined hand movements.

Dataset	Activity	Subjects	Trials	Filter	Feature	Classifier	Accuracy	References
BCI competition III	T1, T2	5	280	Yes		LS-SVM (RBF kernel)	95.72%	[34]
	T1, T2	4	140	Yes		HMM	77.50% +	[35]
	T1, T2	4	200	Yes	CSP	SVM (Gaussian kernel)	77.50%	[36]
	T1, T2	7	100	Yes	CSP/EMD, PCA	KNN	85.8%	[37]
	T1, T2	4	100	Yes		SVM	81%	[29]

Other data sets	T1, T2	4	90	Yes	CSP	SVM (Gaussian kernel)	74.10%	[36]
	T1, T2	1	140	Yes	CSP/ERD-ERS	BSSFO-SVM	97.57%	[25]
	T1, T2		80	Yes	PLS Regression	Based on the decoding principle	64%	[1]
	T1, T2	109	90	Yes	CSP	SUTCCSP	90%	[38]
	T1, T2	4	480	Yes	CSP/ERD-ERS, FFT	LDA, SVM, BPNN	84%+	[39]
	T1, T2	3		Yes	BSS, CSP	SVM	92%	[40]

Epoc	T1, T2	5	120	Yes	CSP/EMD, MIDKRA	PSD, Hjort, CWT, DWT	97.79%	[41]
	T1, T2	8	100	Yes	ERS, ERD	LDA	70.37%	[42]
	T1, T2	2	140	Yes	CSP	Naive Bayes	79%	[43]
	T1, T2	15	40	Yes	Wavelet, PSD, EMD	KNN	91.80%	[44]

**Table 3:** Statistical features extracted using quaternions.

Statistical features	Equation
Mean (*μ*)	$=\frac{\sum \left(\;q_{mod}\right)}{N_{s}}$
Variance (*σ*^2^)	$=\;\frac{{\left(\sum {\left(q_{mod}\right)}^{2}-\mu \right)}^{2}+\sum {\left(q_{mod}\right)}^{2}}{2N_{s}}$
Contrast (con)	$=\frac{\sum {\left(q_{mod}\right)}^{2}}{N_{s}}$
Homogeneity (*H*)	$=\sum \frac{1}{1+{\left(q_{mod}\right)}^{2}}$

**Table 4:** Main modules in QSA and iQSA.

Modules	QSA	iQSA
Sampling window	X	•
Quaternion module	•	•
Classification module	•	•
Boosting technique	X	•
Learning module	X	•
Superposition technique	X	•
Weight normalization	X	•
Majority voting	X	•
Processing type	Batch	Real-time

**Table 5:** Accuracy rate to several data sets of channel blocks.

Data sets	Samples					
	384	320	256	192	128	64
FC5, FC6, P7, P8	74.16	74.13	80.07	81.92	81.60	82.23
F3, F4, FC5, FC6	73.16	74.88	80.23	81.39	81.65	**82.30**
F3, F4, F7, F8	73.48	73.87	79.69	81.74	81.58	82.14
F3, F4, P7, P8	73.47	73.92	80.06	81.68	81.87	**82.33**
AF3, AF4, FC5, FC6	74.17	74.10	80.31	81.82	81.99	82.26

**Table 6:** Table of accuracy rate for iQSA and QSA method with different number samples.

Sample size	QSA (1)	QSA (2)	iQSA (3)
384	40.82%	41.07%	73.16%
320	40.74%	40.29%	74.87%
256	43.56%	43.96%	80.23%
192	42.41%	42.71%	81.39%
128	37.45%	38.28%	81.65%
64	33.31%	33.44%	**82.30**%

**Table 7:** Comparison of performance measures for the decision tree classifier using 64 samples.

Subject	ER	RT	*S*_0_	*S*_1_	*S*_2_	Sp_0_	Sp_1_	Sp_2_
(1)	0.17667	0.82333	0.83000	0.81500	0.82500	0.82000	0.82750	0.82250
(2)	0.18625	0.81375	0.81375	0.79500	0.83250	0.81375	0.82312	0.80437
(3)	0.17542	0.82458	0.85125	0.79000	0.83250	0.81125	0.84188	0.82063
(4)	0.19145	0.80855	0.81923	0.81410	0.79231	0.80321	0.80577	0.81667
(5)	0.20875	0.79125	0.77750	0.79125	0.80500	0.79812	0.79125	0.78437
(6)	0.15792	0.84208	0.86500	0.81875	0.84250	0.83063	**0.85375**	0.84187
(7)	0.18417	0.81583	0.83500	0.79875	0.81375	0.80625	0.82438	0.81687
(8)	0.18333	0.81667	0.82125	0.80625	0.82250	0.81437	0.82188	0.81375
(9)	0.17125	0.82875	0.82875	0.83625	0.82125	0.82875	0.82500	0.83250
(10)	0.16458	0.83542	0.86375	0.84375	0.79875	0.82125	0.83125	**0.85375**
(11)	0.18803	0.81197	0.80641	0.81154	0.81795	0.81474	0.81218	0.80897
(12)	0.15500	**0.84500**	0.83125	0.84000	**0.86375**	**0.85187**	0.84750	0.83562
(13)	0.19542	0.80458	0.83125	0.78250	0.80000	0.79125	0.81563	0.80688
(14)	0.15792	0.84208	0.85000	0.82625	0.85000	0.83813	0.85000	0.83813
(15)	0.19167	0.80833	0.80625	0.79875	0.82000	0.80937	0.81312	0.80250
(16)	0.19458	0.80542	0.82500	0.76875	0.82250	0.79563	0.82375	0.79688
(17)	0.16292	0.83708	0.85000	0.83500	0.82625	0.83063	0.83813	0.84250
(18)	0.17137	0.82863	0.84615	0.81154	0.82821	0.81987	0.83718	0.82885
(19)	0.15875	0.84125	0.84000	0.83250	0.85125	0.84187	0.84562	0.83625
(20)	0.18250	0.81750	0.81500	0.80375	0.83375	0.81875	0.82438	0.80937
(21)	0.31250	0.68750	0.65625	0.68625	0.72000	0.70312	0.68812	0.67125
(22)	0.16708	0.83292	0.86250	0.82125	0.81500	0.81812	0.83875	0.84188
(23)	0.18917	0.81083	0.82375	0.81625	0.79250	0.80437	0.80813	0.82000
(24)	0.16958	0.83042	0.84250	0.81500	0.83375	0.82438	0.83813	0.82875
(25)	0.17500	0.82500	0.84079	0.83947	0.79474	0.81711	0.81776	0.84013
(26)	0.19583	0.80417	0.80750	0.80250	0.80250	0.80250	0.80500	0.80500
(27)	0.16500	0.83500	0.83000	0.82750	0.84750	0.83750	0.83875	0.82875
(28)	0.20083	0.79917	0.78500	0.79625	0.81625	0.80625	0.80063	0.79062
(29)	0.18947	0.81053	0.83421	0.79474	0.80263	0.79868	0.81842	0.81447
(30)	0.16083	0.83917	0.85875	0.83250	0.82625	0.82937	0.84250	0.84563
(31)	0.17875	0.82125	0.83375	0.80625	0.82375	0.81500	0.82875	0.82000
(32)	0.19083	0.80917	0.82125	0.81125	0.79500	0.80312	0.80812	0.81625
(33)	0.19333	0.80667	0.82125	0.79750	0.80125	0.79937	0.81125	0.80937
(34)	0.18917	0.81083	0.80250	0.82000	0.81000	0.81500	0.80625	0.81125
(35)	0.16333	0.83667	**0.86500**	0.82750	0.81750	0.82250	0.84125	0.84625
(36)	0.16833	0.83167	0.83250	0.82250	0.84000	0.83125	0.83625	0.82750
(37)	0.18833	0.81167	0.83125	0.81000	0.79375	0.80188	0.81250	0.82063
(38)	0.17000	0.83000	0.81125	**0.86750**	0.81125	0.83937	0.81125	0.83938
(39)	0.19083	0.80917	0.82125	0.80375	0.80250	0.80313	0.81187	0.81250

*Mean*	*0.18247*	*0.81753*	*0.82533*	*0.81071*	*0.81656*	*0.81363*	*0.82095*	*0.81802*

STD	0.02544	0.02544	0.03451	0.02796	0.02404	0.02315	0.02667	0.0291

**Table 8:** Comparison results.

Subject	iQSA	FDCSP	MEMD-SI-BCI	SR-FBCSP
(1)	0.8543	0.9166	0.9236	0.8924
(2)	0.8296	0.6805	0.5833	0.5936
(3)	0.8273	0.9722	0.9167	0.9581
(4)	0.8509	0.7222	0.6389	0.7073
(5)	0.7879	0.7222	0.5903	0.7810
(6)	0.8284	0.7153	0.6736	0.6998
(7)	0.8172	0.8125	0.6042	0.8824
(8)	0.8466	0.9861	0.9653	0.9532
(9)	0.8425	0.9375	0.6667	0.9192

*Average*	*0.83164*	*0.8294*	*0.7292*	*0.8207*

Std	0.02054	0.1238	0.1581	0.1302

**Table 9:** Friedman test ranking results.

Algorithm	Ranking
iQSA	15.3333
FDCSP	15.5556
SR-FBCSP	16.5556
MEMD-SI-BCI	26.5556

**Table 10:** Li post hoc adjusted *p* values for the test error ranking in Table 9.

Comparison	Adjusted *p* value
iQSA versus datasets	0.00118
iQSA versus SR-FBCSP	0.96717
iQSA versus FDCSP	0.97137
iQSA versus MEMD-SI-BCI	0.70942

**Table 11:** Execution time of iQSA method.

Process	Time in seconds
*iQSA quaternion (1)*	0.2054
*iQSA classification (2)*	0.1035
*iQSA learning (3)*	0.0095

## References

[1] Ofner P., Müller-Putz G. R. (2015). Using a noninvasive decoding method to classify rhythmic movement imaginations of the arm in two planes. *IEEE Transactions on Biomedical Engineering*.

[2] Chai R., Ling S. H., Hunter G. P., Nguyen H. T. Toward fewer EEG channels and better feature extractor of non-motor imagery mental tasks classification for a wheelchair thought controller.

[3] Kim H. S., Chang M. H., Lee H. J., Park K. S. A comparison of classification performance among the various combinations of motor imagery tasks for brain-computer interface.

[4] Wolpaw J. R., McFarland D. J., Neat G. W., Forneris C. A. (1991). An EEG-based brain-computer interface for cursor control. *Electroencephalography and Clinical Neurophysiology*.

[5] Vourvopoulos A., Liarokapis F. Robot navigation using brain-computer interfaces.

[6] Upadhyay R., Kankar P. K., Padhy P. K., Gupta V. K. Robot motion control using Brain Computer Interface.

[7] Wolpaw J. R., Birbaumer N., Heetderks W. J. (2000). Brain-computer interface technology: a review of the first international meeting. *IEEE Transactions on Neural Systems and Rehabilitation Engineering*.

[8] Wolpaw J. R., Birbaumer N., McFarland D. J., Pfurtscheller G., Vaughan T. M. (2002). Brain-computer interfaces for communication and control. *Clinical Neurophysiology*.

[9] Graimann B., Grendan A., Pfurtscheller G. (2010). *Brain-Computer Interfaces, Revolutionizing Human-Computer Interaction*.

[10] Jeannerod M. (1995). Mental imagery in the motor context. *Neuropsychologia*.

[11] Bai O., Huang D., Fei D., Kunz R. (2014). Effect of real-time cortical feedback in motor imagery-based mental practice training. *Neurorehabilitation*.

[12] McFarland D. J., Miner L. A., Vaughan T. M., Wolpaw J. R. (2000). Mu and beta rhythm topographies during motor imagery and actual movements. *Brain Topography*.

[13] Pfurtscheller G., Brunner C., Schlögl A., Lopes da Silva F. H. (2006). Mu rhythm (de)synchronization and EEG single-trial classification of different motor imagery tasks. *NeuroImage*.

[14] Aghaei A. S., Mahanta M. S., Plataniotis K. N. (2016). Separable common spatio-spectral patterns for motor imagery BCI systems. *IEEE Transactions on Biomedical Engineering*.

[15] Gao L., Cheng W., Zhang J., Wang J. (2016). EEG classification for motor imagery and resting state in BCI applications using multi-class Adaboost extreme learning machine. *Review of Scientific Instruments*.

[16] Schlögl A., Lee F., Bischof H., Pfurtscheller G. (2005). Characterization of four-class motor imagery EEG data for the BCI-competition 2005. *Journal of Neural Engineering*.

[17] Trad D., Al-Ani T., Jemni M. (2016). Motor imagery signal classification for BCI system using empirical mode decomposition and bandpower feature extraction. *Broad Research in Artificial Intelligence and Neuroscience (BRAIN)*.

[18] Choi K., Cichocki A. (2008). *Control of a Wheelchair by Motor Imagery in Real Time*.

[19] Millán J. D. R., Renkens F., Mouriño J., Gerstner W. (2004). Noninvasive brain-actuated control of a mobile robot by human EEG. *IEEE Transactions on Biomedical Engineering*.

[20] Lotte F., Congedo M., Lécuyer A., Lamarche F., Arnaldi B. (2007). A review of classification algorithms for EEG-based brain–computer interfaces. *Journal of Neural Engineering*.

[21] Batres-Mendoza P., Montoro-Sanjose C. R., Guerra-Hernandez E. I. (2016). Quaternion-based signal analysis for motor imagery classification from electroencephalographic signals. *Sensors*.

[22] Hamilton W. R. (1847). On quaternions. *Proceedings of the Royal Irish Academy*.

[23] Almonacid M., Ibarrola J., Cano-Izquierdo J.-M. (2016). Voting Strategy to Enhance Multimodel EEG-Based Classifier Systems for Motor Imagery BCI. *IEEE Systems Journal*.

[24] He L., Hu D., Wan M., Wen Y., Von Deneen K. M., Zhou M. (2016). Common Bayesian Network for Classification of EEG-Based Multiclass Motor Imagery BCI. *IEEE Transactions on Systems, Man, and Cybernetics: Systems*.

[25] Suk H.-I., Lee S.-W. (2013). A novel bayesian framework for discriminative feature extraction in brain-computer interfaces. *IEEE Transactions on Pattern Analysis and Machine Intelligence*.

[26] Bermúdez I Badia S., García Morgade A., Samaha H., Verschure P. F. M. J. (2013). Using a hybrid brain computer interface and virtual reality system to monitor and promote cortical reorganization through motor activity and motor imagery training. *IEEE Transactions on Neural Systems and Rehabilitation Engineering*.

[27] Naeem M., Brunner C., Leeb R., Graimann B., Pfurtscheller G. (2006). Seperability of four-class motor imagery data using independent components analysis. *Journal of Neural Engineering*.

[28] Wang L., Xu G., Wang J., Yang S., Guo M., Yan W. Motor imagery BCI research based on sample entropy and SVM.

[29] Schiatti L., Faes L., Tessadori J., Barresi G., Mattos L. Mutual information-based feature selection for low-cost BCIs based on motor imagery.

[30] Abdalsalam M. E., Yusoff M. Z., Kamel N., Malik A., Meselhy M. Mental task motor imagery classifications for noninvasive brain computer interface.

[31] Prakaksita N., Kuo C.-Y., Kuo C.-H. Development of a motor imagery based brain-computer interface for humanoid robot control applications.

[32] Obermaier B., Neuper C., Guger C., Pfurtscheller G. (2001). Information transfer rate in a five-classes brain-computer interface. *IEEE Transactions on Neural Systems and Rehabilitation Engineering*.

[33] Scherer R., Müller G. R., Neuper C., Graimann B., Pfurtscheller G. (2004). An asynchronously controlled EEG-based virtual keyboard: Improvement of the spelling rate. *IEEE Transactions on Biomedical Engineering*.

[34] Siuly S., Li Y. (2012). Improving the separability of motor imagery EEG signals using a cross correlation-based least square support vector machine for brain-computer interface. *IEEE Transactions on Neural Systems and Rehabilitation Engineering*.

[35] Solhjoo S., Nasrabadi A. M., Golpayegani M. R. H. Classification of chaotic signals using HMM classifiers: EEG-based mental task classification.

[36] Park C., Looney D., Ur Rehman N., Ahrabian A., Mandic D. P. (2013). Classification of motor imagery BCI using multivariate empirical mode decomposition. *IEEE Transactions on Neural Systems and Rehabilitation Engineering*.

[37] Hurtado-Rincon J., Rojas-Jaramillo S., Ricardo-Cespedes Y., Alvarez-Meza A. M., Castellanos-Dominguez G. Motor imagery classification using feature relevance analysis: An Emotiv-based BCI system.

[38] Park C., Cheong-Took C. C., Mandic D. P. (2014). Augmented complex common spatial patterns for classification of noncircular EEG from motor imagery tasks. *IEEE Transactions on Neural Systems and Rehabilitation Engineering*.

[39] Tang Z., Sun S., Zhang S., Chen Y., Li C., Chen S. (2016). A brain-machine interface based on ERD/ERS for an upper-limb exoskeleton control. *Sensors*.

[40] Choi K., Cichocki A. Control of a Wheelchair by Motor Imagery in Real Time.

[41] Arias-Mora L., López-Ríos L., Céspedes-Villar Y., Velasquez-Martinez L. F., Alvarez-Meza A. M., Castellanos-Dominguez G. Kernel-based relevant feature extraction to support Motor Imagery classification.

[42] Dharmasena S., Lalitharathne K., Dissanayake K., Sampath A., Pasqual A. Online classification of imagined hand movement using a consumer grade EEG device.

[43] Stock V. N., Balbinot A. Movement imagery classification in EMOTIV cap based system by Naïve Bayes.

[44] Mann-Castrillon D. M., Restrepo-Agudelo S., Areiza-Laverde H. J., Castro-Ospina A. E., Duque-Munoz L.

[45] Janota A., Šimák V., Nemec D., Hrbček J. (2015). Improving the precision and speed of Euler angles computation from low-cost rotation sensor data. *Sensors*.

[46] Kotsiantis S. (2014). A hybrid decision tree classifier. *Journal of Intelligent & Fuzzy Systems: Applications in Engineering and Technology*.

[47] White R. L. (2008). Astronomical applications of oblique decision trees. *AIP Conference Proceedings*.

[48] Elnaggar A. A., Noller J. S. (2010). Application of remote-sensing data and decision-tree analysis to mapping salt-affected soils over large areas. *Remote Sensing*.

[49] Breiman L. (1996). Bagging predictors. *Machine Learning*.

[50] Freund Y., Schapire R. E. Experiments with a new boosting algorithm.

[51] Jiralerspong T., Liu C., Ishikawa J. Identification of three mental states using a motor imagery based brain machine interface.

[52] Fuster J. M. (2000). Prefrontal neurons in networks of executive memory. *Brain Research Bulletin*.

[53] Wang J., Feng Z., Lu N. Feature extraction by common spatial pattern in frequency domain for motor imagery tasks classification.

[54] Gaur P., Pachori R. B., Wang H., Prasad G. A multivariate empirical mode decomposition based filtering for subject independent BCI.

[55] Shenoy H. V., Vinod A. P., Guan C. Shrinkage estimator based regularization for EEG motor imagery classification.

[56] Blankertz B., Dornhege G., Krauledat M., Müller K.-R., Curio G. (2007). The non-invasive Berlin brain-computer interface: fast acquisition of effective performance in untrained subjects. *NeuroImage*.

[57] Rodríguez-Fdez I., Canosa A., Mucientes M., Bugarín A. STAC: a web platform for the comparison of algorithms using statistical tests.

